# Another Brick in the Cell Wall: Biosynthesis Dependent Growth Model

**DOI:** 10.1371/journal.pone.0074400

**Published:** 2013-09-16

**Authors:** Adelin Barbacci, Marc Lahaye, Vincent Magnenet

**Affiliations:** 1 Biopolymers Interactions Assembly UR 1268 (BIA), Institut National de la Recherche Agronomique (INRA), Nantes, France; 2 Laboratoire des sciences de l'ingnieur, de l'informatique et de l'imagerie (ICube), Université de Strasbourg, UMR CNRS 7357, Illkirch, France; Centrum Wiskunde & Informatica (CWI) & Netherlands Institute for Systems Biology, The Netherlands

## Abstract

Expansive growth of plant cell is conditioned by the cell wall ability to extend irreversibly. This process is possible if (i) a tensile stress is developed in the cell wall due to the coupling effect between turgor pressure and the modulation of its mechanical properties through enzymatic and physicochemical reactions and if (ii) new cell wall elements can be synthesized and assembled to the existing wall. In other words, expansive growth is the result of coupling effects between mechanical, thermal and chemical energy. To have a better understanding of this process, models must describe the interplay between physical or mechanical variable with biological events. In this paper we propose a general unified and theoretical framework to model growth in function of energy forms and their coupling. This framework is based on irreversible thermodynamics. It is then applied to model growth of the internodal cell of *Chara corallina* modulated by changes in pressure and temperature. The results describe accurately cell growth in term of length increment but also in term of cell pectate biosynthesis and incorporation to the expanding wall. Moreover, the classical growth model based on Lockhart's equation such as the one proposed by Ortega, appears as a particular and restrictive case of the more general growth equation developed in this paper.

## Introduction

Plant growth implies cell divisions and irreversible expansion of cell wall s. For the latter to occur, two concomitant conditions are required. The first one is the cell wall mechanical deformation in response to the cell turgor pressure build up. The latter results from the aquaporins regulated flow of water in the vacuole driven by osmotic gradient [1], [2]. The second condition is the cell capacity to synthesize, export and incorporate other *bricks* at the inner face of the cell wall to maintain its integrity [3], [4] and to cause its mechanical relaxation. This chemically mediated deformation consists in the reorganization of load-bearing cross-links between the different bricks in the cell wall and in the creation of new ones by the incorporation of new elements.

The structure, organization and dynamics of the load-bearing network remain open questions. The constitutive bricks in the growing plant cell wall consist mainly in three groups of polysaccharides: cellulose micofibrils embedded in a matrix of hemicelluloses and pectins and some structural proteins. Cellulose microfibrils orientation regulates the expansion anisotropy by promoting cell growth along the perpendicular fibers direction [5] in relation with local stress field [6], [7]. Since the 199’s and the “tethered network” [8], xyloglucan (XG), the main growing cell wall hemicellulose hydrogen bounded to cellulose is considered as the main load-bearing network. The extensibility of the cell wall is then controlled by proteins (expansin) and wall-bound enzymes (xyloglucan endotransglycosylase) which dissociate and reorganize the load-bearing cross-links. This chemorheological process [9], [10] promotes the creep of the cell wall. Apart from hemicellulose, the cell wall matrix is composed of pectin. It is made of homogalacturonan (HG), rhamnogalacturonan I (RGI) and II (RGII) rich polysaccharides which ionic interactions and hydrogen bonds play roles in cell-cell adhesion, in the regulation of cell wall porosity and mechanical properties [11]. The major pectic polysaccharide, HG, is secreted in a highly methylesterified form that is later on selectively de-esterification by pectin-methylesterase (PME) [12]. Such modification determines HG cross-links formation via calcium ions that were recently revealed to play an equivalent load-bearing role in substitution of the hemicellulose-cellulose network in an Arabidobsis double mutant *xxt1/xxt2* [13] depleted in XG [14]. Such results reactivated debates on the “tethered network” model [3], [14], [15].

Usually, plant cell growth is investigated from a biophysical point of view in the light of models based on Lockhart's equation [16] (equivalent to Bingham's model) written:
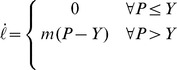
with 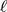 the length, 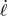 the time derivative of the length (i.e. the growth rate), 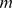 a factor controlling the longitudinal irreversible wall extensibility, 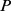 the turgor pressure and 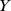 the yield threshold. This equation describes the cell as a non-newtonian fluid, which irreversibly flows when a pressure is applied above a critical yield value. Lockhart's equation describes only the irreversible deformation of the cell and cannot model stress/pressure relaxation and elastic deformation [17]. Thus Ortega [18] proposed a model similar to Maxwell-Bingham equation to account for reversible deformation:



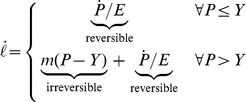
with 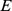 the longitudinal volumetric Young modulus and 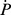 the time derivative of the turgor pressure.

The Lockhart/Ortega's model describes growth rate as a function of cell wall properties modelled initially by the empirical parameters 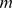, 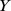, 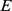. Experiments demonstrated that none of the parameters of Ortega's equation are constant (*e.g.* [17], [19], [20]). At least, the time dependency of each empirical parameter 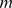, 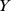, 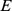 need to be considered. This means that some physical, chemical and biological mechanisms are not described by this equation.

Numerous models have been developed to relate the latter missing mechanisms and to balance the almighty role of turgor pressure. Relationships between load-bearing network and cell wall mechanical properties received specific attention. The Wall-gen software [21] allows the computation of the mechanical properties of the wall in function of interacting cellulose-hemicellulose cross-links. Veytsman and Cosgrove [22] proposed a modified Lockhart's equation to explore relationships between mechanical and chemical energies and to *predict right “trends” in the composition dependency of the cell wall yielding*. They found that the concentration of glucans and cellulose determined the yield threshold and not the strength of hydrogen bonding. The deposition of new material and their kinetics were not addressed by this thermodynamical approach. Dyson and Jensen [23] have modeled the cell wall as a sheet of viscous fibre-reinforced fluid and studied the impact of dynamical changes in its material properties and passive microfibrills reorientation on cell elongation. The effect of proteins such expansin and other enzymes activities was addressed by Pietruszka [24] who introduced time and spatial dependency in Lockhart's parameters. Dyson et al. [25] proposed a dynamical model of hemicellulose cross-links in an expanding wall incorporating strain enhanced breakage and enzyme mediated cross-links kinetics. According to this vision, the yield threshold in Lockhart's equation appeared as dependent of the rate of cross-links breakage over cross-links elongation. The feedback between cell elongation and deposition of polymers in pollen tubes was addressed by Kroeger et al. [26] who considered turgor pressure not only as the driving force but also as a regulator [27]. Rojas et al. [10] proposed a model coupling deposition of new material and mechanical deformation to capture complex pollen tube morphogenesis and to highlight the impact of deposition.

**Figure 1:**
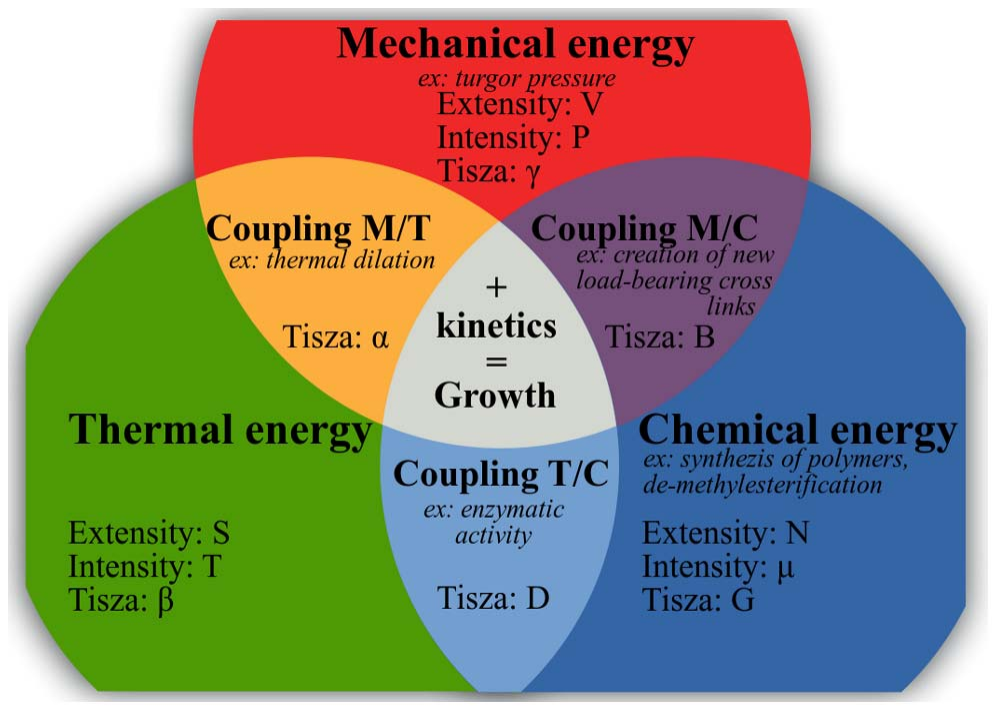
Summary of the theoretical framework. Biological growth is the resulting effect of 3 forms of energy and their coupling (noted M/T, M/C and T/C with M for Mechanical, T for Thermal and C for Chemical). For each energy, each couple of intensive and extensive variables is linked by one component of Tisza's matrix (defined in Eq. 5). An example of the function of each form of energy and coupling is provided.

In light of these efforts, the aim of this paper is to propose a general and unified theoretical framework to model growth considering the deformation due to turgor pressure and the chemorheological process occurring in the cell wall. The idea is to consider growth as a the result of the coupling effect between different forms of energy: mechanical, chemical and thermal (Fig. 1). Mechanical energy is provided by turgor pressure and by stored energy in the load-bearing network regardless of its nature [28]. Chemical energy, refers to the synthesis of new polymers and their possible modifications by enzymes. Thermal energy particularly regulates all enzymatic mechanisms involved in cell wall biosynthesis and modifications. The coupling effect between mechanical and chemical energy reflects chemorheological processes and describes the chemically mediated load-bearing changes, the incorporation of new bricks of polymer at the inner face of the cell wall, the effect of the amorphous matrix on cellulose-hemicellulose network accessibility and enzymes or polymers diffusion [29], the cell wall mechanical relaxation. The coupling effect between chemical and thermal energy account for the enzyme activity whereas the coupling between thermal and mechanical energy for the thermal dilation.

In the next section, we present the general theoretical approach based on an axiomatic thermodynamics proposed by Callen [30] extended in the case of non-equilibrium by Cunat [31]. This theoretical approach is then declined to model the internodal cell growth of *Chara corallina*. This Charophycean alga is a member of the closest relatives of lands plant [32]. Its cell wall peculiarity is the synthesis and incorporation of pectic HG as mostly non-methylesterified structures [33] that readily cross link via calcium [32], [34]. For this model, experimental data were extracted from the work of Proseus and Boyer [34] emphasizing the coupling effects between the mechanical, thermal and chemical energy by changing manually turgor pressure and temperature while measuring their effects on growth.

### General Theoretical Framework

#### Steady state equations

The starting point of the present approach is the axiomatic thermodynamics initially developed by Callen [30]. In his contribution, Callen assumes the existence of a potential function called *internal energy* (noted 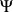), containing all the information of a system and depending *a priori* on all independent extensive variables of the system. Let us recall that a variable is called extensive if, in a composite system, the value of the variable for the whole system is equal to the sum of this variable on every sub-system. In the general case the extensive variables involved in the growth process are: the volume of the representative element 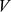 associated with the mechanical energy, the entropy 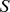 associated with the thermal energy and the quantities of chemical species 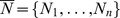 such as enzymes, growth factors and polysaccharides involved in the load-bearing network build-up and reorganization. The latter are associated with the chemical energy (Fig. 1). The internal energy is written:
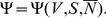
(1)


The differentiation of Eq. 1 defines the intensive variables associated with each extensive variable and by consequence to each form of energy (Fig. 1). The dependency to extensive variables is omitted to lighten equations.
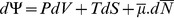
(2)with:
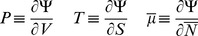
(3)where 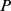 is the turgor pressure, 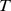 is the temperature and 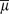 are the chemical potentials (Fig. 1). In the case of the cell, 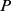 is an internal pressure with a positive sign.

The differentiation of the intensive parameters Eq. 2 leads to the most general form of the constitutive equations written in matrix form:
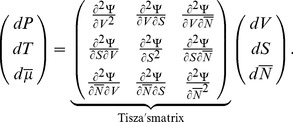
(4)


The matrix linking extensive to intensive parameters is called Tisza's matrix. It is symmetrical since 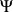 satisfies the Maxwell conditions on integrability involving the second order derivatives of 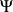. Tisza's matrix has to be positive definite. In the general case, the coefficients of Tisza's matrix are functions of the extensive variables. Eq. 4 can be rewritten in the lighter form:
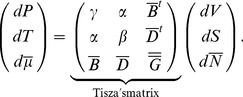
(5)


Tisza's matrix describes all the different forms of energy involved in the biological growth process and their coupling. 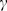 (respectively 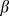 or 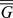) links the extensive to intensive variable corresponding to the mechanical (respectively thermal or chemical) energy. 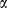, the thermal dilation, describes the coupling effect between mechanical and thermal energies. 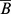, accounting for the chemorheological process, describes the coupling effect between mechanical and chemical energies. 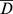, describing the thermal sensitivity of biochemical processes, refers to the coupling effect between the thermal and the chemical energies (Fig. 1). 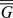 describes the interactions between the different biochemical reactions relative to the synthesis, the assembly of polymers, enzymatic activities….

A very important property of the Tisza matrix is that its components are interrelated via the extensity property of the energy potential. Indeed, Callen [30] demonstrated that if we suppose the internal energy 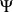 extensive, then Gibbs-Duhem's relationships exist:
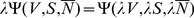
(6)by differentiation of Eq. 6 with respect to 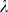 and by taking 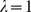, we get:
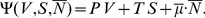
(7)Hence,



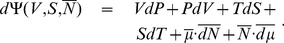
(8)By identification with Eq. 2, Gibbs-Duhem relation is deduced:
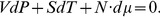
(9)


By injecting the relation Eq. 5 into Eq. 9 and collecting the terms in 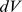, 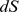 and 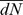 we obtain Gibbs-Duhem relationships written in matrix form
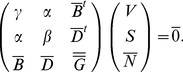
(10)


In practice, these relationships may be useful to express unknown components of Tisza's matrix from known ones.

The key aspect of the present framework is to extend the validity of Eq. 1 outside equilibrium. This assumption has already been formulated by Cunat [31] and gave rise to several successful studies on polymers (e.g. [35]). However, Eq. 5 revealing coupling effects of the different forms of energy does not contain information on the relaxation kinetic of the system back to its equilibrium after a mechanical or thermal solicitation. Thus, an integration of kinetic equations is necessary to supplement the previous thermodynamic equations.

#### Kinetic equations

Internal reorganizations of the cell wall can be modeled by chemical reactions involving the chemical species 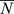 and 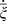, the chemical fluxes (or degrees of reactions) associated with chemical reactions. As an illustration, we consider the chemical reaction presented in Table 1 linking 4 species denoted 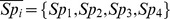 with quantities 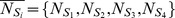. If the species are weighted by stoichiometric ratios 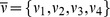 then for the initial time 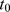 and the time 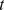, the following quantities 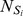 are obtained in function of 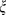 (Table 1). This degree of reaction can describe the entire state of the reaction with the quantity of reagent at 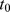 and stoichiometric ratios known. When stoichiometric ratios are equal to one, the degree of reaction is the number of mole produced or consumed by the reaction at the current time. More generally, we introduce the degrees of reactions 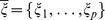 and the affinities 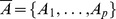 driving the process (also called generalized non-equilibrium forces) given by:

**Table 1:** Example of a chemical reaction linking 4 species 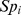 weighted by stoichiometric ratios 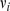.

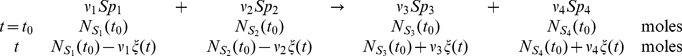

The degree of reaction 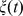 describes the entire state of the reaction if the quantity of reagent at 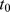 and stoichiometric ratios are known.



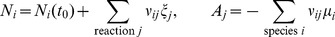
(11)or in matrix form:



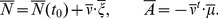
(12)In Eq. 11, 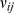 stands for the stoichiometric coefficient of the 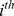 species in the 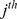 reaction, taken negative if the species is a reagent and positive otherwise.

Rewriting Eq. 5 accounting for Eq. 11 yields:
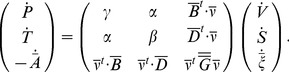
(13)


In general, in the context of irreversible thermodynamics the relation of chemical fluxes can be written in the form [30], [36]:
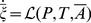
(14)with 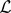 a vector of functions of the intensities (pressure, temperature and affinities) that must be experimentally determined.

As we assume that 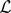 is only a linear function of affinities 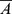 (i.e. 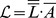 with 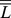 a constant matrix) then the third vectorial equation of Eq. 13 rewrites:
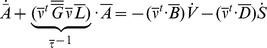
(15)and highlights the definition of the characteristic times 
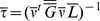
 for the system to reach back equilibrium after a mechanical or thermal solicitation, the latter being driven by the right hand side of Eq. 15. In this sense, it can be seen that the submatrix 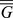 of the Tisza's matrix partially governs the damping behavior described by the constitutive equations (Eq. 13 and Eq. 14). In the more general case where 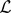 may depend on 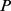 and 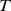 (Eq. 14), non linearities of mechanical or thermal kind can be taken into account, symbolically 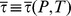


In the next section, a realistic case of growth is treated with the general framework presented in this paragraph. More precisely, Eq. 13 and Eq. 14 were used to model the growth of the internodal cell of *C. corallina*.

## Results and Discussion

### Modeling Growth of *Chara Corallina*

Proseus and Boyer have experimentally demonstrated, by supplying externally pectate, that a chemical mechanism, called “pectate cycle”, controls the growth rate of the internodal cell of *C. corallina* [37]. By changing externally the turgor pressure and the temperature while measuring growth increment [34], the authors also highlighted that the “pectate cycle” is pressure dependant and not only controlled growth but account as well for an efficient mechanism allowing to “store” growth for a while during low pressure period. Data used below to model the cell growth of *C. corallina*, have been extracted from the latter work [34].

#### Phenomenological aspects

**Figure 2:**
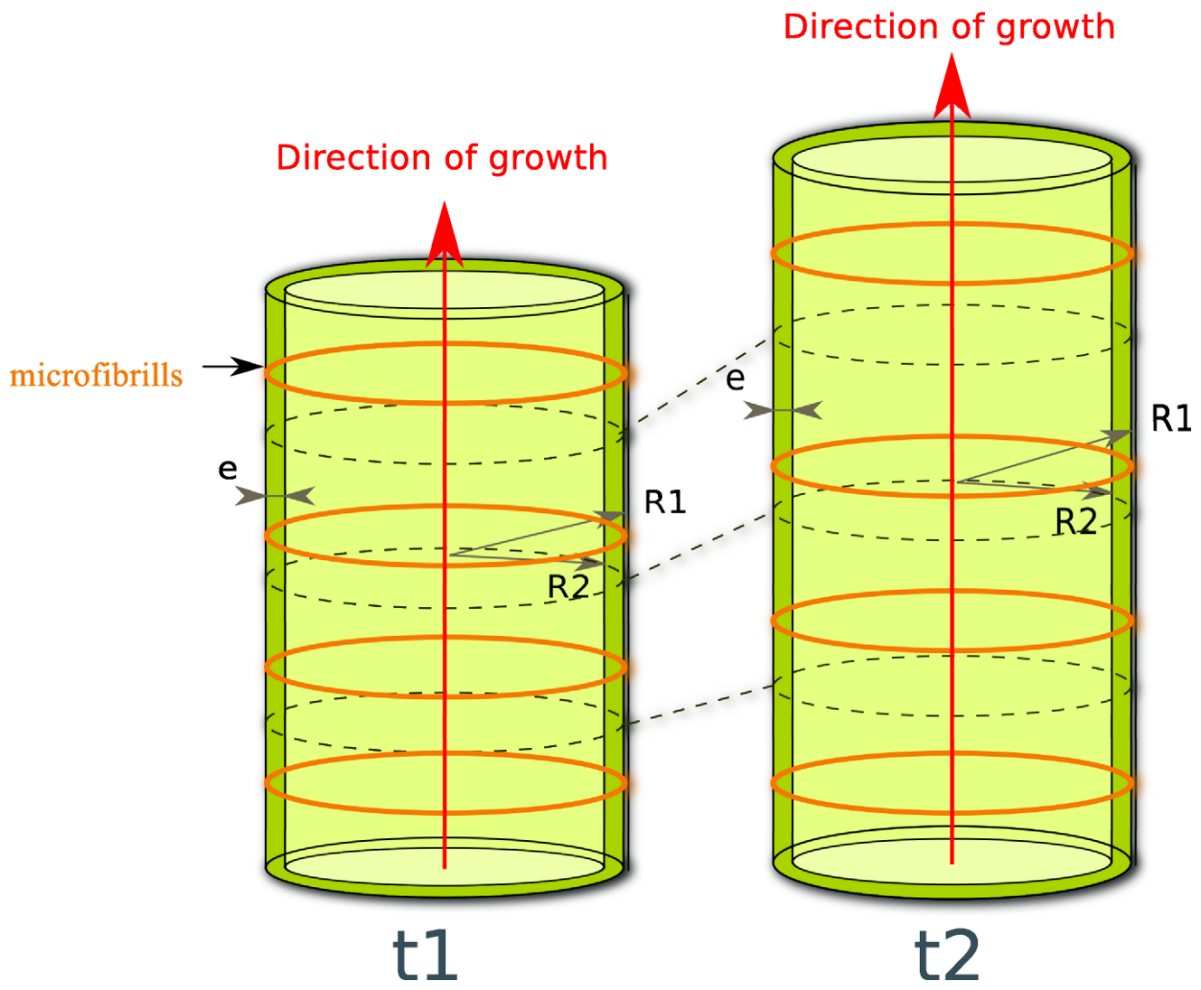
“Pectate cycle” (adapted from [34]). Turgor pressure 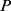 (left) creates a tensile stress in the cell wall. The bounding distance between calcium and pectate increases with pressure inducing a higher mobility of calcium. A non-optimal temperature 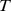 (centre) can reduce the number of free pectate produced by the alga (“U” denotes an unknown reagent introduced in section). When the temperature is optimal for pectate synthesis then free pectate are produced. When 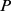 and 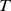 allow growth (right), free pectate are bound with calcium to form new cell wall and involve its mechanical relaxation.

*C. corallina* cell wall is composed of a high amount of mainly non-methylesterified HG pectin (pectate) and a small amount of XG and cellulose [33], [34], [38], [39]. The 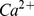-pectate links constitute the main load-bearing network in the wall during growth. Incorporation of new pectate results in the cell wall mechanical relaxation. In this particular case, wall relaxation is a non-enzymatic process and is linked to a temperature and pressure dependent “pectate cycle” (Fig. 2 adapted from [34]). At a given turgor pressure, the created tensile stress in the cell wall increases the calcium mobility (Fig. 2 left) by enlarging bound distances and thus, weakening calcium and pectate bonds. The temperature affects the quantity of free pectate synthesized (Fig. 2 center in which “U” denotes an unknown reagent introduced in the next section). Thus, when temperature and turgor pressure are not limiting factors, free pectates can be incorporated in the extending cell wall and chelate calcium to create new bonds (Fig. 2 right). The creation and the breakage of 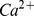-pectate bounds occurs simultaneously providing a wall loosening mechanism [10] driving the growth.

Despite the non-enzymatic bonding process, the extension and the renewal of the cell wall is similar to terrestrial plant such as pollen tubes [10], [32].

**Figure 3:**
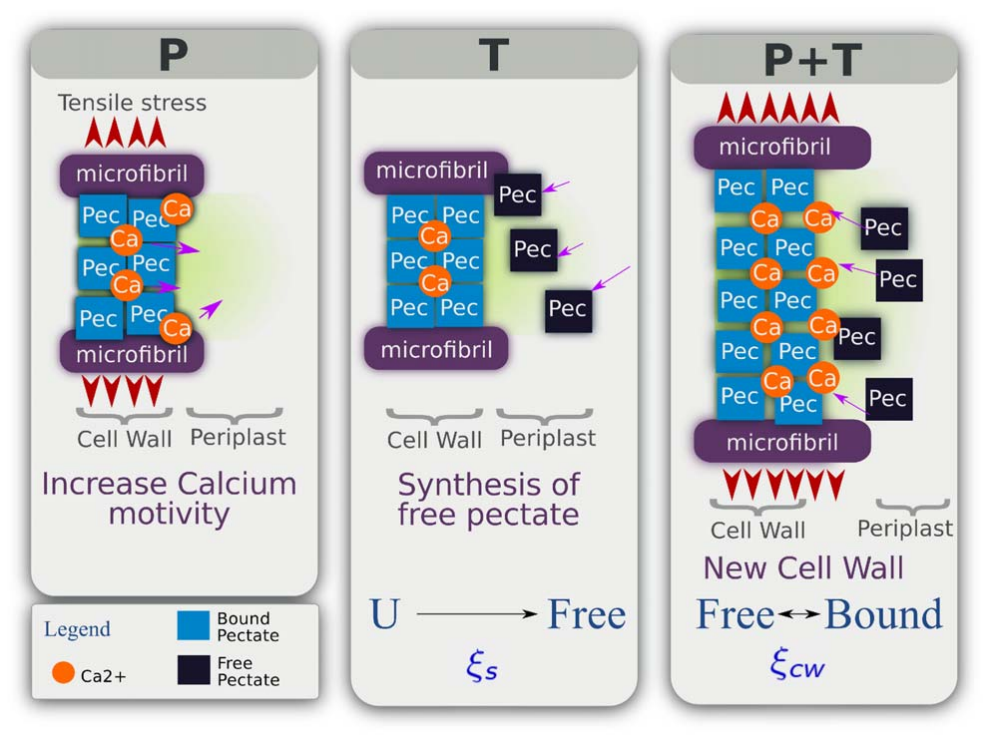
Diffuse and anisotropic growth of the internodal cell of *C. corallina*. Growth occurs in the longitudinal direction at every point of the cell wall whereas the thickness (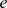) and the internal 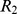 and external 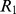 radii of the cell remain constant in time (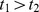).

Growth of the alga is diffuse, anisotropic (Fig. 3) and not restricted to the cell tip. The direction of growth is perpendicular to the direction of the cellulose microfibrils axis in the cell wall *i.e.* along the longitudinal direction of the cell. The cell wall thickness and the transverse section area (noted 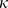) are considered constant with time (which is true at least on the time range of the experiment).

#### Representative volume

On a thermodynamic point of view, the representative volume that need to be considered in order to use the proposed framework is that of the cell wall. The latter is assumed to be under an homogeneous stress state so that the Cauchy stress tensor may be decoupled into two contributions, as it is classically done for porous media (see e.g. [40]):
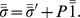



In this equation, 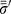 stands for the so-called effective stress, that is the stress exerted on the solid part of the cell wall, while the contribution 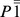 is due to the fluid bathing it. If an infinitesimal change occurs on 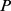, that is 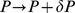, the mechanical response of the cell wall will be characterized by a change on the effective stress 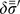 so that the total stress 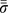 keeps satisfying the boundary conditions on the cell and the equilibrium equation 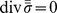 (in this case reducing to 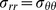). In the cylindrical coordinates 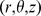 associated with the cell, we can consider that the Cauchy stress tensor has the form
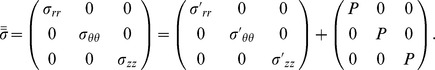



If we assume that the cell grows only in its longitudinal direction, a change in the turgor pressure implies only a deformation 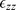, and we have 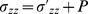. If no longitudinal stress is applied to the boundary of the cell, that is 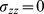, we obtain 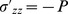, and 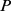 becomes the only mechanical intensive variable describing the solid part of the cell wall. Its extensive counterpart is the length 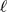, up to a factor 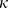 being equal to the section of the cell wall. Note that in this first approach, the radial components 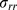 and 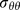 have not been modeled. Finally, the system to which our thermodynamic framework is applied is the solid part of the cell wall, which is submitted to the turgor pressure 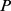.

#### Constitutive equations

Based on the previous section, extensive variables describing the system are the volume of the cell wall 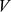 proportional to the length of the cell 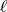 (the transverse section 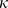 of the cell wall 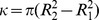 remains constant Fig. 3), the entropy 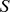, the quantity of free pectate 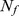, bound pectate 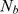 *i.e.* pectate in the cell wall and 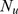 reagents involved in the synthesis of free pectate. 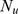 is an arbitrary variable used to describe the complex synthesis processes of pectate. The internal energy then reads:
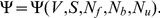
(16)


The differentiation of Eq. 16 leads to a similar equation to Eq. 2 and to a similar form of constitutive equation presented in Eq. 5.

In the specific case of *C. corallina* cell growth, Tisza's matrix is simplified thanks to the latter experimental evidences. We assumed that the elongation rate was only function of bound pectate (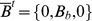), that the assembling process was not function of temperature (
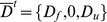
) and that the number of free pectate synthesized is not dependent on the amount of bound pectate (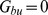 in 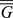). The thermal dilation 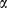 is neglected because of the small range of variation of the temperature. Hence, the final form of the constitutive equations for the growing *C. corallina* cell is:
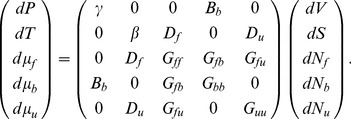
(17)


The Gibbs-Duhem relationships (Eq. 10) allow expressing 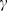, 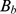, 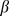 and 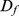 in function of the chemical coupling matrix 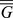:
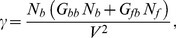
(18)

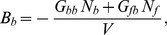
(19)

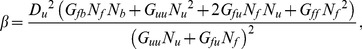
(20)

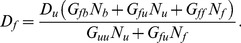
(21)


Thus, the number of unknown parameter of Tisza's matrix decreases from 10 to 6.

#### Kinetics

To model growth of *C. corallina* internodal cell, two chemical reactions are considered in a simple manner because the exact reactions are unknown. Such chemical model and approximation have already been used to model the mechanical expansion of pollen tubes cell wall [10].

The first one describes the synthesis of new pectate. A simple way to model the reaction is to suppose that an unknown reagent (noted 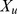) produces free pectate:
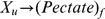
(22)


The sign of the affinity of the reaction gives the direction of the reaction 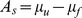. If 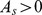 then free pectate are produced and unknown reagents are consumed. *A priori*, 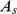 is positive. The second reaction describes the cell wall elongation *i.e.* the incorporation of new pectate within the existing cell wall by creation of 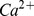-pectate load-bearing cross links.
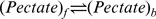
(23)


The direction of the reaction is still determined by the sign of the reaction affinity 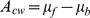. *A priori*, 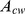 is positive. Considering Eq. 22 and Eq. 23 the quantity of chemical components of the growing cell wall is defined as follows.
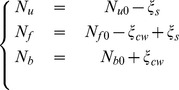
(24)with 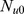, 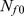, 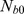 the initial quantities of unknown reagents, free and bound pectate. In addition 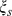 and 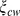 are the degrees of the two reactions *i.e.* the number of mole of synthesized and bound pectate. In the context of irreversible thermodynamics such as treated by Prigogine and Kondepudi [36], the relation between chemical fluxes and intensities (Eq. 14) can be written



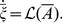
(25)This form depends only on the affinity of reactions and allows exploring the chemical part of the energy and its coupling. As concerns kinetics laws, we consider, in this first approach, a first order Taylor's development of the general relations presented in Eq. 25, viz.
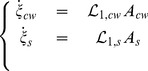
(26)in which 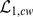 and 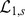 are constant coefficients. Eq. 17 then becomes:
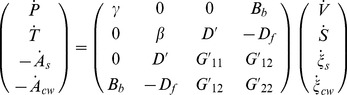
(27)with



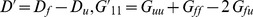


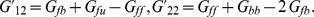



Note that the matrix of Eq. 27 is still symmetrical.

#### Growth equation

By expressing the rate of entropy in Eq. 27.
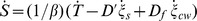



Eq. 27 becomes.
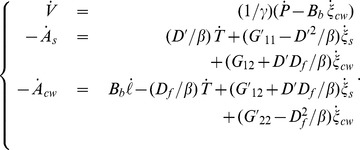



By replacing expressions of 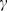, 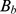, 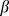, 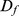 by Eq. 18, Eq. 19, Eq. 20, Eq. 21 and 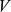 by 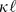 we obtain the growth equation system written in matrix form:
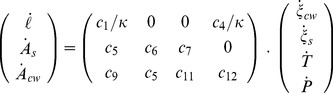
(28)with 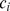 function of Tisza's matrix coefficients and extensities (expressions of 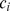 are given in Table 2) and rates of reactions 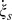, 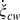 expressed in kinetic equations (Eq. 26).

**Table 2:** Expressions and signs obtained after fit of 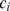 involved in growth equation (Eq. 28).

	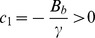
	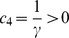
	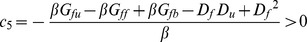
	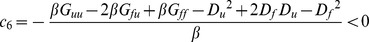
	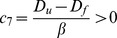
	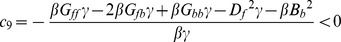
	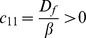
	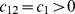

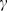
, 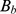, 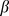 and 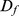 known and expressed by Eq. 18 to Eq. 21.

**Figure 4:**
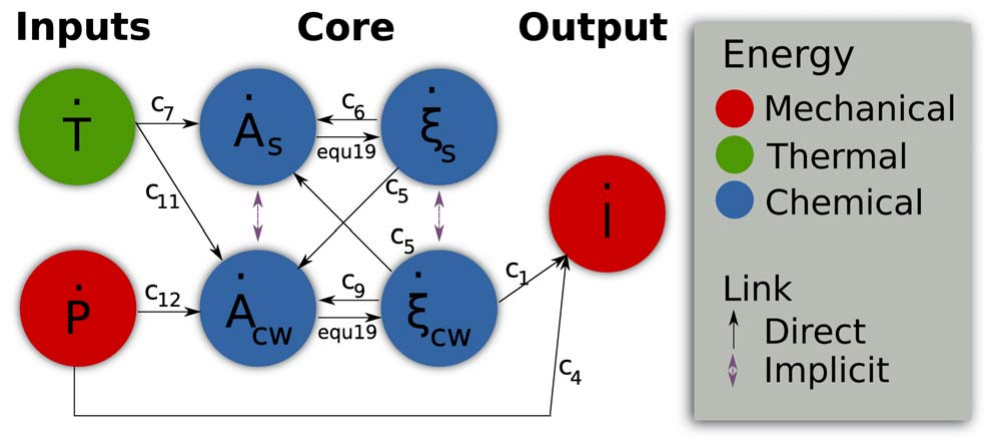
Rational of the growth equation system (Eq. 28). Pressure and temperature rates are the inputs. All the variables of the core, relative to the chemical part of the energy (i.e. the synthesis and assembly reactions) are coupled by growth equation coefficients 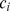 (Eq. 28) and by kinetic equations (Eq. 26). Loops in the core part, model regulation processes. The growth rate, the output, is the resulting effect of the chemical activity modeled by the core and of the pressure rate. When pressure and temperature are constant, growth increment is determined by the kinetics of cell wall creation 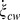.

The growth equation system (Eq. 28) is constituted by non-linear differential equations. In broad outlines, it describes growth increment 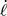 as always dependent on the synthesis rate of 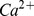-pectate cross link 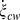 and, when 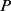 is not constant also dependant of a deformation induced by the turgor pressure change. In other word, the two conditions needed for cell growth (a tensile stress due to pressure and the capacity to incorporate new bricks at the inner face of cell wall to lengthen it and to relax the tensile stress) are modeled naturally by such thermodynamics framework. A rational of the system is presented on Fig. 4. The core of the model is composed by the two chemical reactions relative to the synthesis of free pectate and their assembly in the cell wall. The rate of pectate incorporation 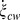 drives directly the growth 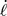 (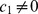), influences the synthesis of free pectate 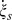 (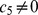) and its future evolution 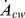 (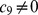). The kinetics of chemical reactions are dependant on changes of turgor pressure and temperature (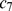,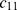,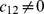). Thus, when temperature and pression are not limiting factors and do not vary (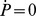 and 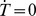), growth occurs depending on the rate of pectate incorporation. This incorporation of new bricks in the wall involves its mechanical relaxation [10]. Pressure rate has a double role. It can influence growth rate directly by deforming the wall (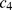 in Eq. 28 and on Fig. 4) but is also a regulator of growth since it influences directly (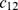 in Eq. 28) and implicitly (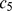 in Eq. 28) the kinetics of chemical response of the equation system (loops in core graph Fig. 4). This complex role of pressure which is a necessary and not sufficient growth condition, has already been observed and studied on pollen tubes [10], [26].

#### Result of the fit

**Figure 5:**
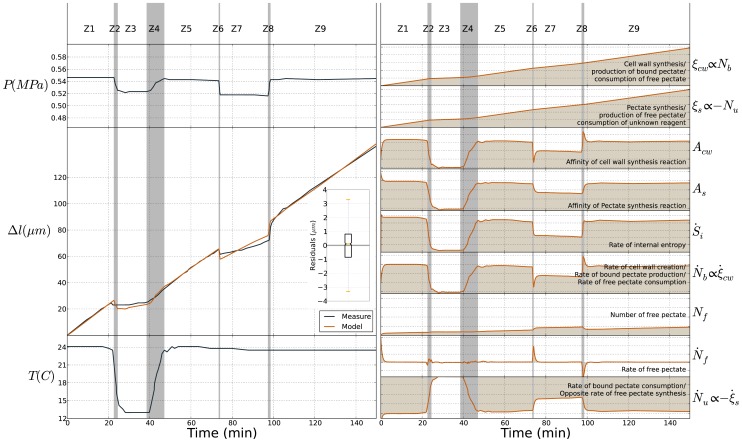
Results of the fit of the Eq. 28. Left panel: in black turgor pressure 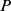, temperature 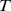 (externally manipulated) and resulting growth increment 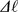 (data extracted from [34]). In orange, result of the fit. Inset depicts the distribution residuals. Right panel: in orange results given by the model. From top to bottom: degrees of cell wall assembly 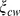 reaction (proportional to the number of bound pectate 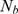), free pectate synthesis 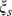 (proportional to the number of unknown reagent used to produce free pectate 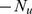), chemical affinities of the two reactions 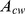 and 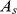, rate of internal entropy 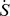, temporal evolution of quantity of pectate in the cell wall 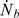 (proportional to the speed of creation of new cell wall), number of free pectate 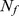, temporal evolution of free pectate 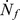, temporal evolution of unknown reagent 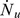 (proportional to the speed of free pectate synthesis 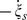). The 9 zones noted 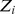 (colored in light grey) are corresponding to the different test set of the experiment. Orange shade between 0 and the curve indicate the sign.

The value obtained for each parameter is presented in Table 3 and the result of the fit is depicted on Fig. 5 (depicted in orange in the Fig. 5, data extracted from Proseus and Boyer [34] are depicted in black). The agreement between length increment modeled and experimental data is quite good (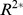 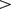 0.998). Tisza's matrix obtained with the set of parameters is positive definite since all the eigenvalues are positive (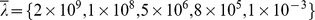).

**Table 3:** Parameters of the model.

Name	Value	Unit	Dimensions	Adjusted	Sensitivity Rank
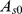	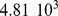	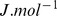	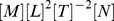	yes	1
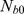	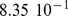	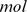	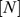	yes	2
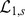	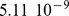	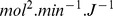	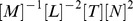	yes	3
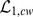	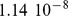	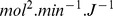	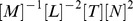	yes	4
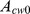	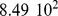	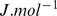	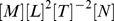	yes	5
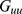	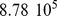	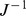	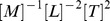	yes	6
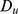	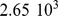	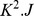	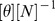	yes	7
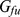	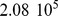	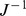	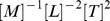	no	8
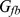	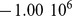	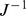	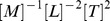	no	9
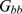	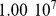	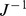	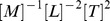	no	10
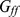	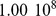	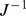	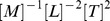	no	11
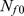	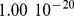	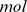	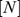	no	12
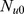	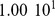	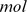	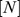	no	13

Value, unit and dimensions of the growth model parameters.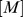 mass dimension, 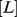 length dimension, 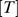 time dimension, 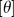 temperature dimension, 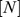 chemical quantity dimension. 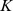 Kelvin, 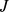 Joule, 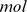 mole, 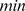 minute.

The amount of pectate presents in the cell wall and synthesized is globally growing (Fig. 5 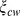, 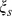).

The temporal evolution of the internal entropy, defined as the sum of the temporal evolution of entropy of the two reactions [36].
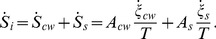
(29)is growing too (Fig. 5 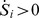). The direction of reactions accounting for synthesis and new material incorporation is never inverted during the experiment since the sign of 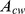 and 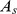 is always positive. When both pressure and temperature decrease (Fig. 5 Z2), the number of free pectate grows (Fig. 5 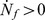) and stops when pressure and temperature reach the low level (Fig. 5 Z3). Growth does not occur during Z3 since the flux of cell wall produced is close to 0 (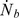 and 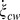 Fig. 5 Z3). As the pectate synthesis reaction is stopped (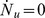 Fig. 5 Z3) the number of free pectate remains constant (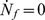 Fig. 5 Z3) until pressure and temperature reach their initial levels (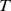 Fig. 5 Z4). During this period, free pectate stocked and produced (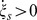 Fig. 5 Z4) are consumed to produce new cell wall (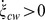 Fig. 5 Z4) and the number of free pectate tends towards its initial value (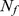 Fig. 5 Z4). The growth increment increases with the same rate than during the first period (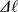 Fig. 5 Z1) but on a different trajectory. Alternatively, when only the pressure decreases (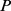 Fig. 5 Z6), the two reactions slow down but do not stop (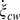 and 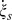 positive but smaller than during Z5). The number of free pectate grows during the low pressure time range (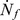 Fig. 5 Z7) and when pressure is set to its initial level (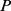 Fig. 5 Z8) the stock of these pectate is quickly consumed to produce cell wall (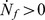 Fig. 5 Z8). The growth rate reaches its initial value but, unlike the Z3 period, on the same trajectory as Z5 when temperature and pressure are at the normal level.

Deviations between the model and the experiment are mostly explained by the simple form of kinetics equations (Eq. 26) used to model chemical fluxes. Other forms of kinetics should be formulated in the light of new experiments.

The results given by the proposed model, despite its qualitative aspect due to the lack of quantitative data, describe accurately the interplay between cell elongation and the “pectate cycle” by modification and extension of the 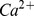-pectate load-bearing network in function of pressure and temperature in *C. corallina*.

Analysis of signs of 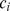 obtained after the fit of Tisza's matrix parameters (Table 2) allows extracting the global scheme of *C. corallina* internodal cell growth. A variation of pressure or of the rate of pectate incorporation causes a growth variation of the same sign (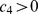,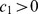). The creation of new 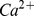-pectate tends to stop the assembly process (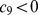) but this tendency is counteracted either by the amount of free pectate available (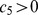) or a raise of pressure (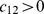) or temperature (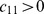). The more pectate are linked by chelation, the more free pectate are synthesized (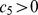) but the synthesis of new pectate can be stopped by a reduction of temperature (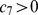). This joint fluctuation is modified by amount of free pectate (variation of 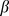 Eq. 20 and 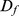 Eq. 21 in 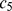 in function of 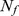). This dependency of the rate of incorporation of free pectate with the cell wall is an important mechanism which allows the algal cell to *store* growth when pressure is low for a certain length of time and to *resume* growth when pressure reaches a non limiting value [34].

These results are in total agreement with the experimental evidences found by Proseus and Boyer [34] and sketched on Fig. 2.

#### Back to Lockhart/Ortega's equation

To model growth of *C. corallina* cell, the form of the function describing the rate of reactions Eq. 14 was chosen as proposed by Prigogine [36] and Callen [30], in order to explore the relations between growth and cell wall remodeling and led to Eq. 25. To develop a more physical approach, focusing mostly on the mechanical part of the energy, another form of the Eq. 14 can be used. If temperature is omitted and if the cell wall synthesis and assembly are coarsely described, we can assume the rate of reactions expressed as in Lockhart's equation.
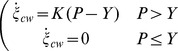
(30)with 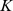 a positive constant, 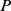 the pressure and 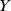 a threshold pressure. The Eq. 30 models the turgor pressure as driving the growth when the pressure is over the threshold 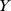, its role of regulator disappears. This new form of chemical fluxes and the constitutive equations (Eq. 30) imply that the growth rate becomes independent of other growth equations describing the affinities (Eq. 28) since only mechanical quantities 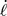 and 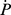 remain. The growth increment is then modeled by a unique equation:



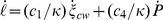
(31)

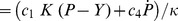
(32)


By identification with Ortega's equation.
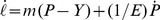
(33)


we obtain.
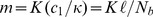
(34)

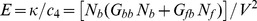
(35)with 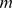 the longitudinal irreversible wall extensibility and 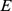 the longitudinal volumetric elastic modulus. Hence, we obtain a cell wall chemical composition dependent expression of the two Ortega's equation parameters. Others expressions can be established by a different use of Gibbs-Duhem relationships (Eq. 10).

**Figure 6:**
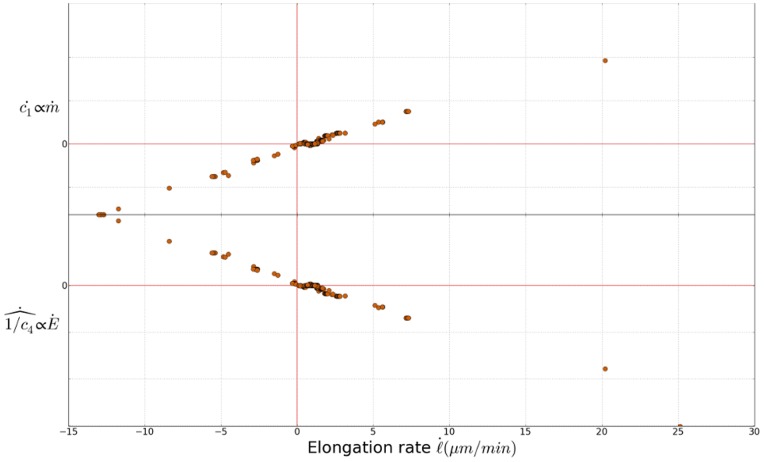
Comparison with Ortega's model. Instantaneous time variation of 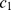 and 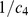. The evolution of these parameters in function of growth rate agrees with results in [17].

The magnitudes of parameters 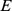 and 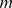 have been estimated *in vivo* for fungal single-cell sporangiophores of *Phycomyces blakesleeanus* and algal single-celled internodes of *C. corallina* (in [17]). These studies established that 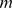 increases and 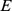 decreases with the elongation rate. This result is coherent with ours (Fig. 6) since we observed the same variations for our parameters 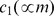 and 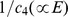.

### Experimental Limits

The efficiency of this approach, despite the flexibility of the equations due to Gibbs-Duhem relationships (Eq. 10), depends on the accessibility of measurements. In particular, some intensities or extensities such as chemical potential 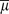 and entropy 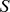 are not measurable. Therefore, predicting pressure as a function of the biochemical behaviour is impossible without assumptions on Tisza's matrix coefficients. To obtain 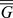, measuring growth triggered by more complex variations of temperature and pressure would be pertinent to obtain a more robust fit. In any case, new experiments focussed on producing quantitative biochemical measurements are required to describe the initial quantity of polymers (Eq. 24) or their relative abundance and kinetics of reaction (*i.e.* the form of 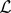 in Eq. 25).

### A Framework for Growth Models

The thermodynamical framework allows considering growth as the result of coupling effect between different forms of energy without restrictive hypothesis. The application of the general growth equation (Eq. 13) to the case of the *C. corallina* cell growth, in spite of its qualitative aspect, can be viewed as the first step towards a more integrative biophysical modeling of growth.

Complex regulation loops, as the one involving turgor pressure and temperature in *C. corallina* cell growth, seems to be naturally described by the present approach due to the strong non linearity of the growth equations. Future modeling of plant growth experiments involving complex regulations such as the ones involved in perceptive mechanisms like mechanoperception [41] or proprioception [42] will allow testing the robustness of our approach.

## Materials and Methods

### Parameters Estimations

The quantitative aspect of the “pectate cycle” is unknown. Consequently, to test our approach, some assumptions were made on the chemical part of the energy to determine the initial values of parameters to fit. Thus, the results of the fit will only be qualitative concerning the “pectate cycle”. The quantity of unknown reagent used by the cell to produce free pectate was supposed unlimited (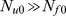). In the same manner, the number of pectates composing the cell wall at the beginning of the experiment was supposed much higher than the number of free pectates (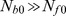). Parameters (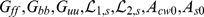) were chosen in order to have the number of pectate synthesized 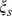 and added to the cell wall 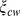 in the same order of size. Signs of 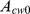 and 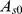 were set positive meaning that at the initial time free pectate was synthesized and free pectate was added to the cell wall. Consequently, the set of 13 parameters to fit is composed per components of the Tisza's matrix, of initial quantities of chemical species involved in the two reactions, and of parameters used to describe the chemical kinetics called 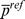 such as
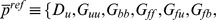


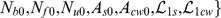



In order to reduce the number of parameters to adjust, a sensitivity analysis on the simulated length 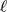 was performed in the vicinity of the parameters set 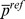. The sensitivity 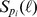 of a given parameter 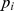 around its value 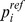 was computed as:
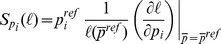


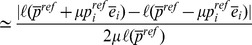
with 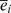 the i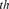 vector of the canonical basis of 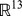 and 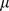 “sufficiently small” (0.01 in practice). More easily, 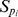 quantifies the relative variation of 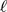 for a relative variation of 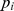 equal to 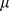.

**Figure 7:**
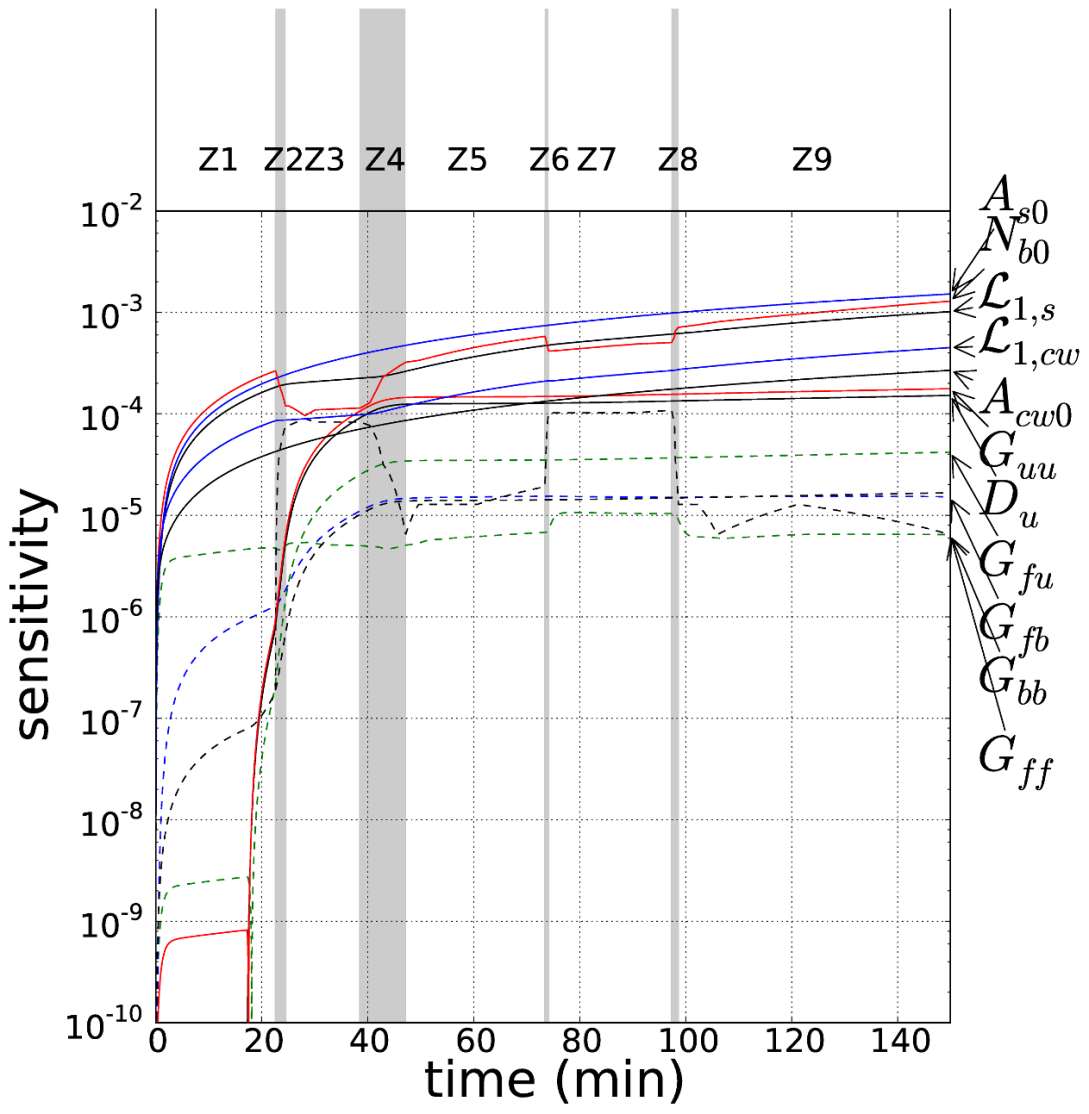
Time evolution of the sensitivity of the model parameters (in logscale). In dashed lines, non-sensitive parameters. On the right, names of the variables refer to parameters defined in the text (Eq. 28). 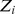
's refer to the test-set of the experiment defined by different pressure and temperature levels (Fig. 5). Note that the sensitivity of the parameter 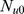 and 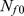 disappears.

The results of the sensitivity test are presented in Fig. 7. Parameters with a sensitivity in the highest decade were chosen for the fit. Thus, the set of parameters to fit was reduced to 7 {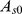,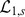,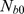,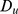,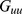,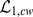,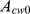} whereas others were kept constant (Table 3).

### Computation of Observable Variables

The vector of observable variables was numerically computed from a time discretization of the following equations:
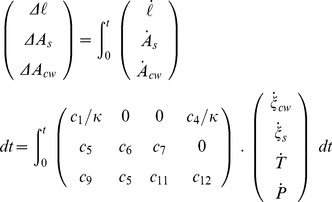
(36)


The inversion of the model was carried out in the least square sense using a Levenberg-Marquardt algorithm. Measured turgor pressure and temperature (extracted from [34] Fig. 5 in purple) were used to determined length increment (Fig. 5 in black). The initial length 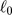 was set to 5 cm and the thickness of the cell wall 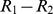 to 5 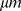 and the inner radius 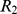 to 2.5 mm (Fig. 3).

### Goodness of Fit


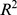
 coefficient has been computed as
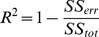
(37)with
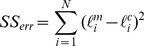
(38)and



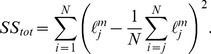
(39)

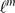
 is the length measured by Proseus and Boyer [34] and 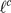 the length computed by the model.

To account for the number of parameters in our model, an adjusted R-squared 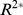 has been computed as:
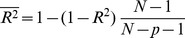
(40)


In our case, the number of points 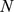 is equal to 1500 and the number of regressors 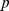 to 13.

## References

[1] Zonia L, Munnik T (2007) Life under pressure: hydrostatic pressure in cell growth and function. Trends in plant science 12: 90–7. 17293155 10.1016/j.tplants.2007.01.006

[2] Dumais J, Forterre Y (2012) Vegetable dynamicks: The role of water in plant movements. Annual Review of Fluid Mechanics 44: 453–478.

[3] Cosgrove DJ (1999) Enzymes and other agents that enhance cell wall extensibility. Annual review of plant physiology and plant molecular biology 50: 391–417. 10.1146/annurev.arplant.50.1.391 11541953

[4] Lerouxel O, Cavalier DM, Liepman AH, Keegstra K (2006) Biosynthesis of plant cell wall polysaccharides - a complex process. Current opinion in plant biology 9: 621–30. 17011813 10.1016/j.pbi.2006.09.009

[5] Baskin TI (2005) Anisotropic expansion of the plant cell wall. Annual review of cell and developmental biology 21: 203–22. 10.1146/annurev.cellbio.20.082503.103053 16212493

[6] Hamant O, Traas J (2010) The mechanics behind plant development. The New phytologist 185: 369–85. 20002316 10.1111/j.1469-8137.2009.03100.x

[7] Boudaoud A (2010) An introduction to the mechanics of morphogenesis for plant biologists. Trends in plant science 15: 353–360. 20427223 10.1016/j.tplants.2010.04.002

[8] Fry SC, Smith RC, Renwick KF, Martin DJ, Hodge SK, et al (1992) Xyloglucan endotransglycosylase, a new wall-loosening enzyme activity from plants. Biochem J 282: 821–828. 1554366 10.1042/bj2820821 PMC1130861

[9] Ray P, Ruesink A (1962) Kinetic experiments on the nature of the growth mechanism in oat coleoptile cells. Developmental biology 4: 377–397.

[10] Rojas ER, Hotton S, Dumais J (2011) Chemically mediated mechanical expansion of the pollen tube cell wall. Biophysical journal 101: 1844–53. 22004737 10.1016/j.bpj.2011.08.016 PMC3192986

[11] Caffall KH, Mohnen D (2009) The structure, function, and biosynthesis of plant cell wall pectic polysaccharides. Carbohydrate research 344: 1879–900. 19616198 10.1016/j.carres.2009.05.021

[12] Pelloux J, Rustérucci C, Mellerowicz EJ (2007) New insights into pectin methylesterase structure and function. Trends in plant science 12: 267–77. 17499007 10.1016/j.tplants.2007.04.001

[13] Cavalier DM, Lerouxel O, Neumetzler L, Yamauchi K, Reinecke A, et al (2008) Disrupting two arabidopsis thaliana xylosyltransferase genes results in plants deficient in xyloglucan, a major primary cell wall component. The Plant cell 20: 1519–37. 18544630 10.1105/tpc.108.059873 PMC2483363

[14] Park YB, Cosgrove DJ (2011) Changes in cell wall biomechanical properties in the xyloglucan-deficient xxt1/xxt2 mutant of Arabidopsis. Plant physiology 158: 465–475. 22108526 10.1104/pp.111.189779 PMC3252101

[15] Thompson DS (2005) How do cell walls regulate plant growth? Journal of experimental botany 56: 2275–85. 16061505 10.1093/jxb/eri247

[16] Lockhart JA (1965) An analysis of irreversible plant cell elongation. Journal of Theoretical Biology 8: 264–275. 5876240 10.1016/0022-5193(65)90077-9

[17] Geitmann A, Ortega JK (2009) Mechanics and modeling of plant cell growth. Trends in plant science 14: 467–78. 19717328 10.1016/j.tplants.2009.07.006

[18] Ortega JK (1985) Augmented growth equation for cell wall expansion. Plant physiology 79: 318–320. 16664396 10.1104/pp.79.1.318 PMC1074876

[19] Cosgrove DJ (1985) Cell wall yield properties of growing tissuel. Plant Physiology: 347–356. 10.1104/pp.78.2.347 PMC1064733 16664243

[20] Moulia B, Fournier M (2009) The power and control of gravitropic movements in plants: a biomechanical and systems biology view. Journal of experimental botany 60: 461–86. 19264759 10.1093/jxb/ern341

[21] Kha H, Tuble SC, Kalyanasundaram S, Williamson RE (2010) Wallgen, software to construct layered cellulose-hemicellulose networks and predict their small deformation mechanics. Plant physiology 152: 774–86. 20007450 10.1104/pp.109.146936 PMC2815898

[22] Veytsman BA, Cosgrove DJ (1998) A model of cell wall expansion based on thermodynamics of polymer network. Biophysical journal 75: 2240–2250. 9788919 10.1016/S0006-3495(98)77668-4 PMC1299898

[23] Dyson RJ, Jensen OE (2010) A fibre-reinforced fluid model of anisotropic plant cell growth. Journal of Fluid Mechanics 655: 472–503.

[24] Pietruszka M (2012) A biosynthesis/inactivation model for enzymatic wlfs or non-enzymatically mediated cell evolution. Journal of theoretical biology 315: 119–27. 23021969 10.1016/j.jtbi.2012.09.016

[25] Dyson RJ, Band LR, Jensen OE (2012) A model of crosslink kinetics in the expanding plant cell wall: yield stress and enzyme action. Journal of theoretical biology 307: 125–36. 22584249 10.1016/j.jtbi.2012.04.035 PMC3414840

[26] Kroeger JH, Zerzour R, Geitmann A (2011) Regulator or driving force? the role of turgor pressure in oscillatory plant cell growth. PloS one 6: e18549. 21541026 10.1371/journal.pone.0018549 PMC3081820

[27] Winship LJ, Obermeyer G, Geitmann A, Hepler PK (2010) Under pressure, cell walls set the pace. Trends in plant science 15: 363–9. 20483654 10.1016/j.tplants.2010.04.005 PMC2999822

[28] Dumais J (2007) Can mechanics control pattern formation in plants? Current opinion in plant biology 10: 58–62. 17140841 10.1016/j.pbi.2006.11.014

[29] Videcoq P, Steenkeste K, Bonnin E, Garnier C (2013) A multi-scale study of enzyme diffusion in macromolecular solutions and physical gels of pectin polysaccharides. Soft Matter 9: 5110.

[30] Callen HB (1985) Thermodynamics and an introduction to thermostatistics. 2nd edition. New York: Wiley, 2nd revise edition, 512 pp.

[31] Cunat C (2001) The dnlr approach and relaxation phenomena. part I historical account and dnlr formalism. Mechanics of Time-Dependent Materials 5: 39–65.

[32] Peaucelle A, Braybrook S, Höfte H (2012) Cell wall mechanics and growth control in plants: the role of pectins revisited. Frontiers in plant science 3: 121. 22685449 10.3389/fpls.2012.00121 PMC3368173

[33] Sørensen I, Pettolino Fa, Bacic A, Ralph J, Lu F, et al (2011) The charophycean green algae provide insights into the early origins of plant cell walls. The Plant journal: for cell and molecular biology 68: 201–11. 21707800 10.1111/j.1365-313X.2011.04686.x

[34] Proseus TE, Boyer JS (2008) Calcium pectate chemistry causes growth to be stored in chara corallina: a test of the pectate cycle. Plant, cell & environment 31: 1147–55. 10.1111/j.1365-3040.2008.01829.x 18507807

[35] Mrabet K, Rahouadj R, Cunat C (2003) An irreversible model for semicristalline polymers submitted to multisequence loading at large strain. Engineering and Science 45: 42–51.

[36] Prigogine I, Kondepudi D (1999) Thermodynamique des moteurs thermiques aux structures dissipatives. Odile Jacob, odile jacob edition.

[37] Proseus TE, Boyer JS (2006) Calcium pectate chemistry controls growth rate of chara corallina. Journal of experimental botany 57: 3989–4002. 17110588 10.1093/jxb/erl166

[38] Proseus TE, Boyer JS (2006) Identifying cytoplasmic input to the cell wall of growing chara corallina. Journal of experimental botany 57: 3231–42. 16893975 10.1093/jxb/erl087

[39] Proseus TE, Boyer JS (2007) Tension required for pectate chemistry to control growth in chara corallina. Journal of experimental botany 58: 4283–92. 18182431 10.1093/jxb/erm318

[40] Coussy O (2004) Poromechanics. Ltd, John Wiley & Sons, 2nd rev ed edition, 312 pp.

[41] Moulia B, Der Loughian C, Bastien R, Martin L, Rodríguez M, et al. (2011) Integrative mechanobiology of growth and architectural development in changing mechanical environments. In: Wojtaszek P, editor, Mechanical integration of plant cells and plants., Berlin, Heidelberg: Springer Berlin Heidelberg, volume 9 of *Signaling and Communication in Plants*, chapter Integrative. 269–302.

[42] Bastien R, Bohr T, Moulia B, Douady S (2013) A unifying model of shoot gravitropism reveals proprioception as a central feature of posture control in plants. Proceedings of the National Academy of Sciences of the United States of America in press. 10.1073/pnas.1214301109 PMC3545775 23236182

